# Sucrose promotes caries progression by disrupting the microecological balance in oral biofilms: an *in vitro* study

**DOI:** 10.1038/s41598-020-59733-6

**Published:** 2020-02-19

**Authors:** Qian Du, Min Fu, Yuan Zhou, Yangpei Cao, Tingwei Guo, Zhou Zhou, Mingyun Li, Xian Peng, Xin Zheng, Yan Li, Xin Xu, Jinzhi He, Xuedong Zhou

**Affiliations:** 1 The state key Laboratory of Oral Diseases, National Clinical Research Center for Oral Diseases, West China Hospital of Stomatology, Sichuan University, Chengdu, Sichuan China; 2 University of Chinese Academy Sciences-Shenzhen Hospital, Shenzhen, China; 3 The Department of Endodontics and the Division of Constitutive & Regenerative Sciences, UCLA School of Dentistry, Los Angeles, CA 90095 USA; 4 Center for Craniofacial Molecular Biology, Herman Ostrow School of Dentistry, University of Southern California, 2250 Alcazar Street, Los Angeles, CA 90033 USA; 5 Clinical Skills Training Center, West China Hospital, Sichuan University, Chengdu, Sichuan China

**Keywords:** Symbiosis, Biofilms, Microbial ecology, Microbiome, Dental caries

## Abstract

Sucrose has long been regarded as the most cariogenic carbohydrate. However, why sucrose causes severer dental caries than other sugars is largely unknown. Considering that caries is a polymicrobial infection resulting from dysbiosis of oral biofilms, we hypothesized that sucrose can introduce a microbiota imbalance favoring caries to a greater degree than other sugars. To test this hypothesis, an *in vitro* saliva-derived multispecies biofilm model was established, and by comparing caries lesions on enamel blocks cocultured with biofilms treated with sucrose, glucose and lactose, we confirmed that this model can reproduce the *in vivo* finding that sucrose has the strongest cariogenic potential. In parallel, compared to a control treatment, sucrose treatment led to significant changes within the microbial structure and assembly of oral microflora, while no significant difference was detected between the lactose/glucose treatment group and the control. Specifically, sucrose supplementation disrupted the homeostasis between acid-producing and alkali-producing bacteria. Consistent with microbial dysbiosis, we observed the most significant disequilibrium between acid and alkali metabolism in sucrose-treated biofilms. Taken together, our data indicate that the cariogenicity of sugars is closely related to their ability to regulate the oral microecology. These findings advance our understanding of caries etiology from an ecological perspective.

## Introduction

Dental caries, one of the most prevalent diseases occurring on tooth hard tissues, is driven by a disequilibrium in the oral microbial community that is termed dental biofilm^1–3^. Dental biofilm is a highly organized polymicrobial structure on tooth surfaces^4^ and is enmeshed in an extracellular matrix whose major component is extracellular exopolysaccharides (EPS)^5^. By metabolizing dietary fermentable carbohydrates, microorganisms within the dental biofilm generate organic acids (e.g., lactic acid). When acid production exceeds the neutralizing capacity of both alkali-producing bacteria and saliva, the low pH caused by acid accumulation within the dental biofilm initiates demineralization of tooth hard tissues^4,6–11^. Meanwhile, the acidic environment favors the growth of acidic/aciduric species but not alkali-producing bacteria, which in return prompts the progression of dental caries and the formation of tooth cavities^2,7,10,11^.

There is a consensus that carbohydrates, especially dietary sugars, determine whether caries develops or not^12^. Three variables of sugar consumption, the amount, frequency and sugar type, are closely related to caries progression^13,14^, as studies showed that individuals frequently taking large amounts of specific sugars experienced greater caries severity relative to those with a lower intake^15,16^. In addition to serving as bacterial metabolism substrates for energy production, sugars also affect the formation and properties of dental biofilms. For example, oral bacteria use sugars to synthesize EPS^17^, while EPS enhance the adherence of biofilm to tooth surfaces^18,19^, mediate biofilm three-dimensional organization and structural integrity^12^, and increase biofilm porosity, thereby facilitating the diffusion of sugars into the inner layer of biofilms^20^. EPS also help build a protective microenvironment for residing bacteria by retarding the diffusion of antimicrobial molecules^21^ and act as an extracellular energy source supporting the survival of microorganisms within the biofilm^21,22^.

Previous studies indicated that the cariogenicities of different sugars were distinct^23^. Among the kinds of dietary sugars, sucrose (C_12_H_22_O_11_) has long been regarded as an “arch criminal” of dental caries^24^. Some early animal studies showed that when animals were fed different sugars at the same concentration, sucrose treatment produced more severe caries lesions than glucose (C_6_H_12_O_6_), fructose (C_6_H_12_O_6_), maize starch ((C_6_H_10_O_5_)n) and amylopectin ((C_6_H_10_O_5_)n)^23,25–27^. Further mechanistic exploration revealed that the prominent cariogenicity of sucrose compared to that of other sugars was partly because this disaccharide was the sole substrate for the synthesis of water-insoluble glucans (the major unit of EPS)^12,17^. Moreover, compared to biofilms formed in the presence of glucose and fructose, biofilms formed in the presence of sucrose had lower concentrations of Ca, P and F^28^, all of which act as mineral reservoirs to prevent demineralization of tooth hard tissues.

Considering that caries is driven by ecological dysbiosis of oral commensal bacteria, some pioneering studies have focused on microbial changes after sucrose supplementation, trying to provide new clues to understand the cariogenicity of sucrose, and they observed some ecological responses in the oral microbial community after sucrose supplementation. For example, Minah *et al*. found that exposure to high concentrations of sucrose (i.e., 10%) stimulated the succession of *Veillonella spp*., *Lactobacillus spp*., *Streptococcus salivarius*, and *Streptococcus mutans* and decreased the levels of *Streptococcus sanguinis*, *Neisseria spp*., and gram-negative anaerobic rods^29^. Another study showed that a sucrose-enriched diet caused an inverse relationship between *S. sanguinis* (a caries-preventing bacteria) and *S. mutans* (a cariogenic bacteria) abundances^30^. However, these studies analyzed only specific/individual species and failed to define the entire oral microbial community shift after sucrose treatment. Moreover, to the best of our knowledge, it remains unclear until now whether the ecological stress imposed by sucrose is greater than that imposed by other sugars.

Therefore, in the present study, we hypothesized that the cariogenicity of sucrose is highly associated with its ability to regulate the oral microecology. To test this hypothesis, we established an *in vitro* saliva-derived multispecies biofilm model, profiled and compared the microbial structure and composition between biofilms exposed to sucrose and two other dietary sugars (i.e., glucose and lactose). By doing so, we aimed to provide new insights from an ecological perspective to understand the cariogenicity of sucrose and caries etiology.

## Results

### Sucrose is a more cariogenic dietary sugar than glucose and lactose

**Figure 1:**
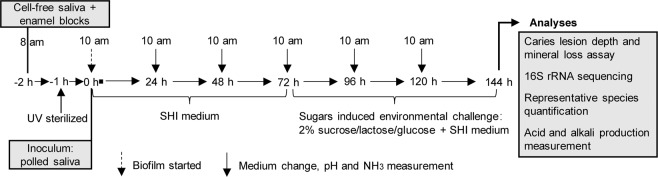
Biofilm preparation and treatment regimen. The saliva inoculum was inoculated in 1.5 ml of SHI medium and cultured without disturbance during the first 24 h to form an initial biofilm community on the enamel block surface. Starting at 72 h, the biofilms were transferred to SHI medium containing different dietary sugars (i.e., 2% sucrose, 2% lactose and 2% glucose) to simulate cariogenic challenges until the endpoint (144 h). The culture medium was changed every 24 h until the end of the experimental period. The control group was saliva derived biofilm cultured with SHI medium only.

**Figure 2:**
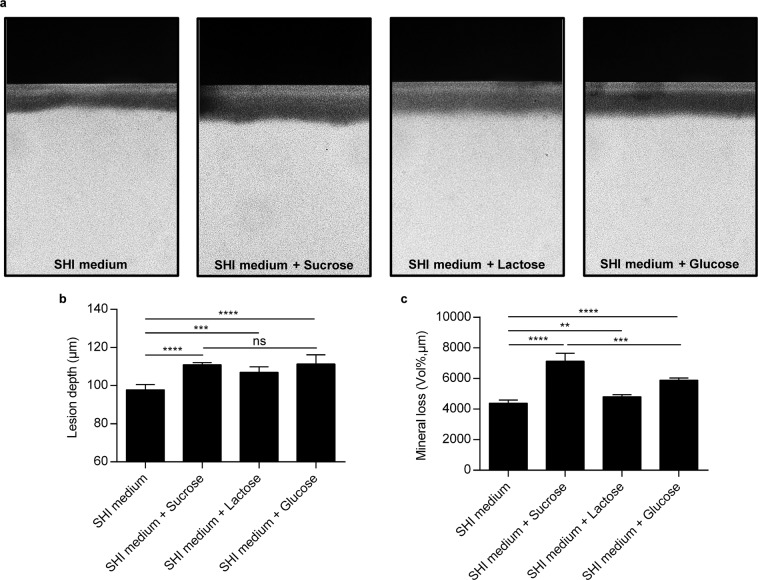
Sucrose-treated biofilms exhibit the most significant demineralization effect on enamel blocks. (**a**) Representative transverse microradiography images of enamel blocks from sucrose, lactose and glucose treated groups at 144 h. Quantification of lesion depth (**b**) and mineral loss (**c**) of enamel blocks at the endpoint. All results are presented as mean ± standard deviation (n = 5; **p < 0.01; ***p < 0.001; ****p < 0.0001; ns, not significant).

To test our assumption that the cariogenicity of sucrose is dependent on its microecology regulation, we first established an *in vitro* saliva-derived multispecies biofilm model (Fig. 1) and tested whether this model could reproduce the *in vivo* finding that sucrose is the most cariogenic dietary sugar^12,28,31–33^. After 3 days of dietary sugar treatment, enamel blocks cocultured with sugar-treated biofilms showed obviously enhanced demineralization compared to blocks from the SHI medium group (Fig. 2). Specifically, sucrose, lactose and glucose significantly increased the demineralized lesion depth and mineral loss of the tooth enamel; however, sucrose showed the strongest demineralization-promoting effect when applied to saliva-derived multispecies biofilms (Fig. 2b,c). Although the lesion depth caused by sucrose, glucose and lactose was similar, the mineral loss caused by sucrose supplementation was significantly higher than that caused by glucose and lactose (Fig. 2b,c).

### Sucrose but not glucose or lactose influences the microbial structure and assembly of the oral microbiota

**Figure 3:**
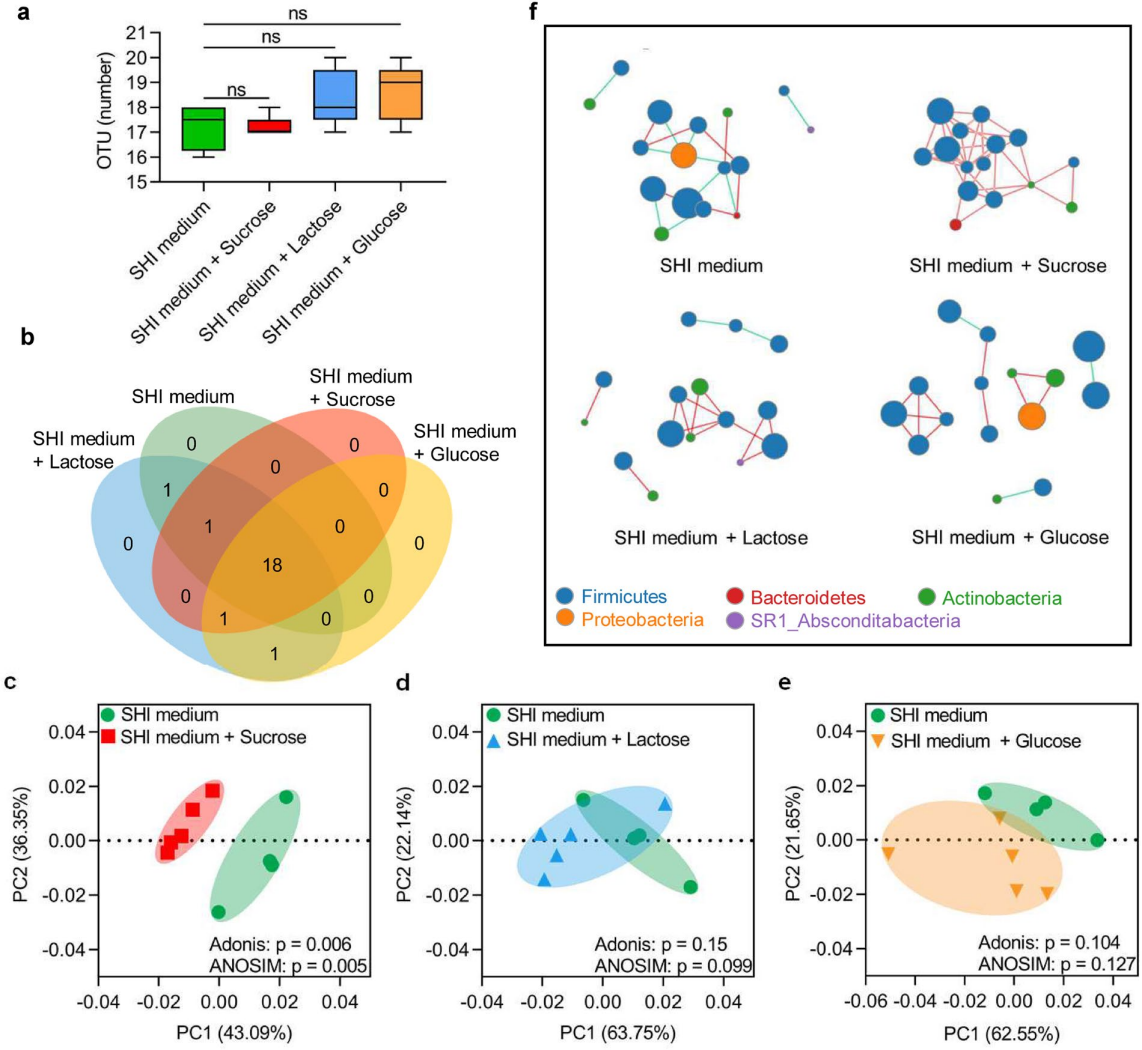
Sucrose influences the microbial structure and assembly of the oral microecology. (**a**) The microbial richness as revealed by OTU number. (**b**) Venn diagram showing shared OTUs among biofilms treated without/with sucrose, lactose and glucose. (**c–e**) The PCoA and dissimilarity analysis (including Adonis and ANOSIM) of polymicrobial oral biofilm among groups. Each dot stands for a biofilm sample. (**f**) Network inferences of microbial relationships in biofilms without/with sucrose, lactose or glucose treatment. Each node represents an OTU, and each edge represents a significant pairwise association. Red lines indicate synergistic relationships while green lines represent antagonistic relationships (n ≥ 4; ns, not significant; OTU, operational taxonomic unit; PCoA, principal coordinates analysis).

The genomic DNA of biofilms was isolated and sequenced to investigate the microbiome shift introduced by sugar supplementation. After preprocessing and filtering, the average amplicon length of the remaining high-quality reads was 518 bp. These reads were assigned to 22 operational taxonomic units (OTUs) at 97% similarity. Microbial richness as revealed by OTU number showed no statistically significant differences among groups (p > 0.05, Fig. 3a). Venn diagram analysis showed that 18 out of 22 OTUs were shared by all groups and that there was no sugar-specific OTU that was exclusively detected in one group (Fig. 3b). By applying weighted UniFrac principal coordinates analysis (PCoA) based on the Bray-Curtis distance (Fig. 3c) and dissimilarity tests, including Adonis and ANOSIM, we found that the overall microbial structure of sucrose-treated biofilms was significantly different from that of biofilms of the control group (Adonis: R^2^ = 0.47, p = 0.006; Adonis: R^2^ = 0.825, p = 0.005). However, both weighted PCoA and dissimilarity tests detected no significant differences in the microbial community structure between the lactose/glucose treatment group and the SHI medium control (Fig. 3d,e). Ecological network analyses at the OTU level were also constructed to better show how microbial community assembly changed after sucrose, glucose and lactose treatments. Although the networks of all sugar treated groups contained 15 nodes (16 nodes in SHI medium group), and all networks were dominated by OTUs belonging to *Firmicutes*, there were more intermodular connections in the network of the sucrose group than in the that of either the glucose or lactose group. More importantly, the antagonistic relationship indicated by the green line between OTUs in the sucrose group was totally lost (Fig. 3f).

### Sucrose introduces the most severe homeostasis disruption between acid-producing and alkali-producing bacteria

**Figure 4:**
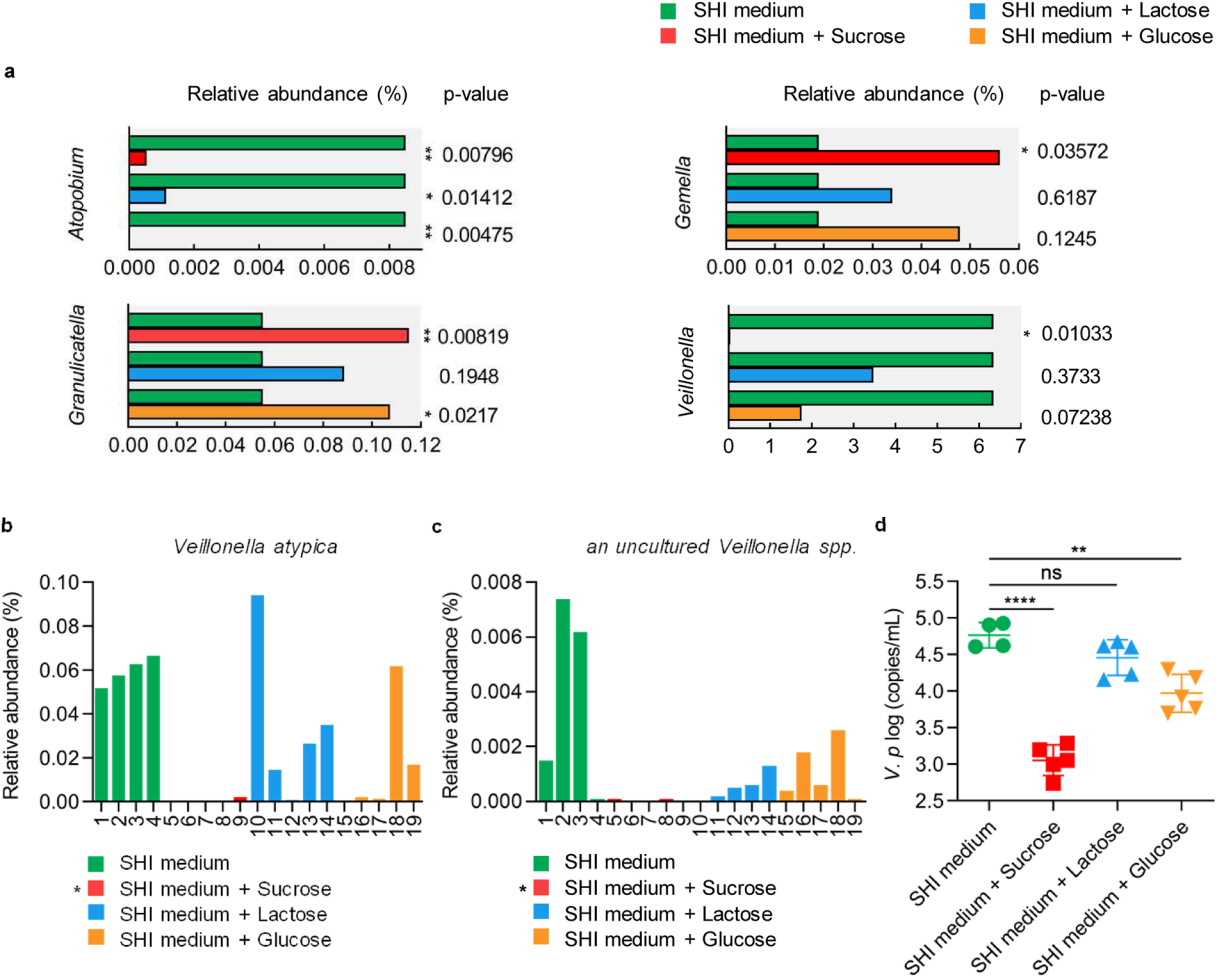
Sucrose significantly decreases the level of bacteria contributing to acid reduction. (**a**) Taxa distribution at the genus level. Only genera showed significantly different distribution were included. (**b**,**c**) Different distributed *Veillonella* species among samples. Each column stands for one sample. (**d**) qPCR quantification of *V. parvula* in saliva derived biofilm without or with sucrose/lactose/glucose treatment. Results were presented as mean ± standard deviation (n ≥ 4; *p < 0.05; **p < 0.01; ****p < 0.0001; ns, not significant; *V. p* = *V. parvula*).

From the sequencing data, we pinpointed the genera that were distributed differently between dietary sugar-treated biofilms and the control biofilm. The distributions of 4 genera were disturbed by sucrose, while only 2 genera showed different distribution patterns between glucose-treated biofilms and the control biofilm (Fig. 4a). Lactose imposed the least pressure on the microbial community, as only 1 genus demonstrated a change in relative abundance (Fig. 4a). Among the 4 genera (*Atopobium, Granulicatella, Gemella* and *Veillonella*) that showed different relative abundances between the sucrose group and the control, *Veillonella* is of particular interest because species belonging to this genus can metabolize lactic acid produced by cariogenic bacteria by converting lactic acid to acetic acid and propionic acid, which are less acidic^34–36^. The genus *Veillonella* changed its distribution in only sucrose-treated biofilms (Fig. 4a). The distributions of species belonging to *Veillonella* were further compared, and we found that the relative abundance of *Veillonella atypica* and an *uncultured Veillonella spp*. significantly decreased in the sucrose-treated group based on sequencing data (Fig. 4b,c). The different distribution pattern of another *Veillonella* species, that is, *V. parvula*, was also confirmed by qPCR since we did not detect this species from the sequencing data, possibly due to low abundance, and the colonization of *V. parvula* showed a similar tendency as other *Veillonella* species (Fig. 4d). Although the bacterial load of *V. parvula* in the glucose-treated biofilms also decreased compared to that of the control biofilm, it decreased less than that of the sucrose group.

**Figure 5:**
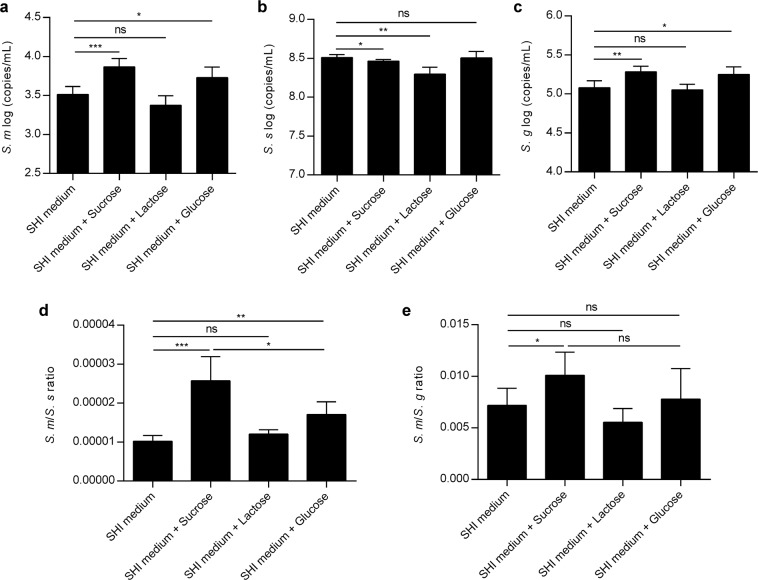
Sugar disrupts the equilibrium between acid- and alkali-producing bacteria. qPCR quantification of *S. mutans* (**a**), *S. sanguinis* (**b**), *S. gordonii* (**c**), and the ratios of *S. mutans*/*S. sanguinis* (**d**) and *S. mutans*/*S. gordonii* (**e**) in biofilm treated without or with sucrose/lactose/glucose. Results were presented as mean ± standard deviation. (n = 5; *p < 0.05; **p < 0.01; ***p < 0.001; ns, not significant; *S. m* = *S. mutans*; *S. s* = *S. sanguinis*; *S. g* = *S. gordonii*).

We further quantified the acid-producing bacteria *S. mutans* and alkali-producing bacteria, including *S. sanguinis* and *Streptococcus gordonii*, within the biofilms using qPCR to determine the homeostasis disruption between acid-producing and alkali-producing bacteria. Sucrose exposure increased the concentrations of *S. mutans* and *S. gordonii* but slightly decreased the concentration of *S. sanguinis*. Biofilms receiving glucose treatment also had higher concentrations of *S. mutans* and *S. gordonii* than the control biofilm, whereas the concentration of *S. sanguinis* was unaffected. Interestingly, lactose had a contrasting effect to glucose, as the levels of *S. mutans* and *S. gordonii* were unchanged; however, the level of *S. sanguinis* decreased (Fig. 5a–c). Since the *S. mutans*/*S. sanguinis* or *S. mutans*/*S. gordonii* ratio is believed to be more closely correlated with dental caries than the load of single acid-producing or alkali-producing bacteria^37–40^, we further compared these ratios (summarized in Fig. 5d,e). Although both sucrose and glucose treatments increased the ratio of acid-producing/alkali-producing bacteria, the *S. mutans*/*S. sanguinis* and *S. mutans*/*S. gordonii* ratios within sucrose-treated biofilms were higher than those within the glucose-treated biofilms. In addition, no significant difference was observed between lactose-treated biofilms and the control group biofilm (Fig. 5d,e).

### Dysbiosis of acid-producing and alkali-producing bacteria results in disequilibrium between acid and alkali metabolism, directly contributing to caries progression

**Figure 6:**
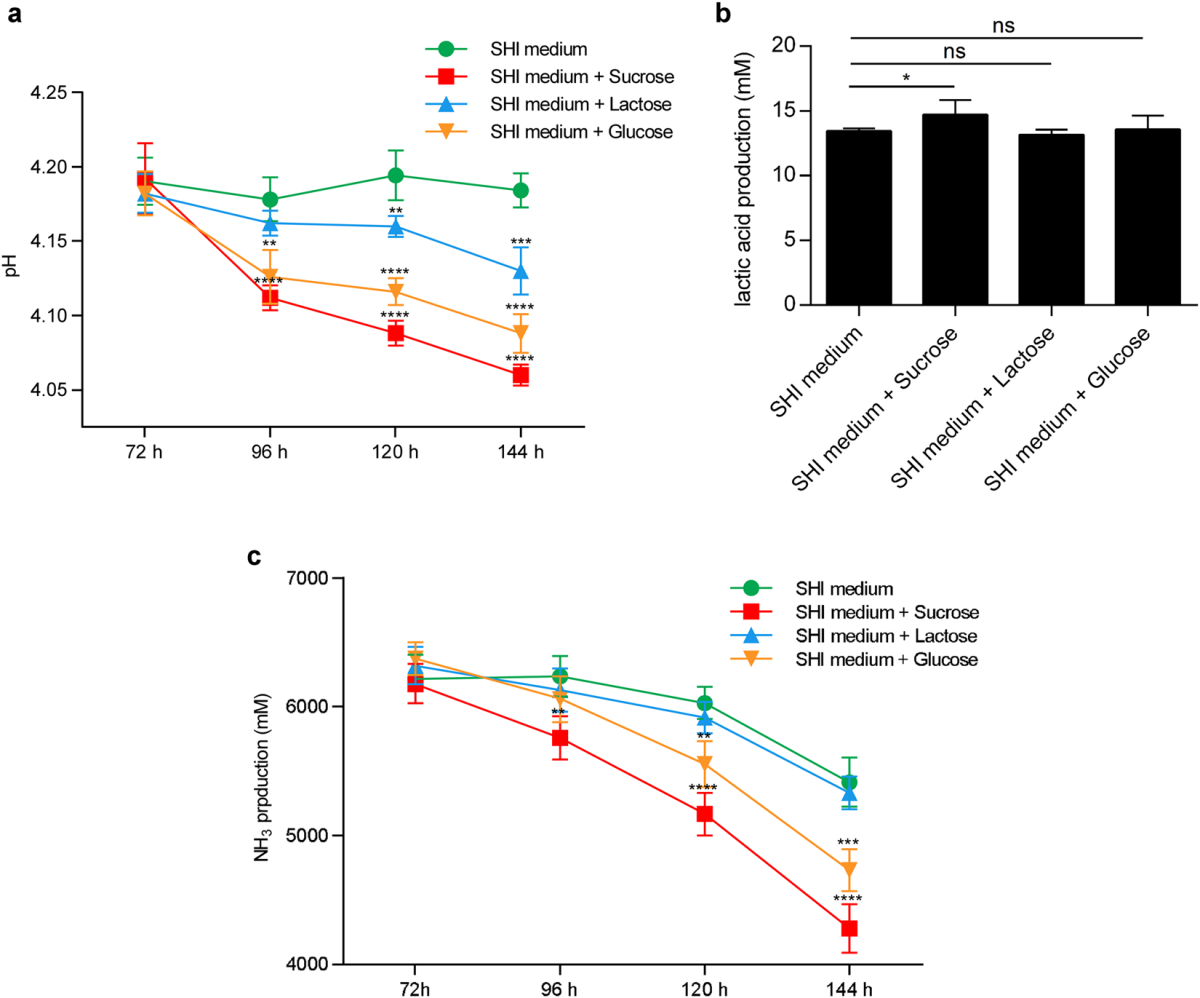
Sugar exposure increases the cariogenicity of saliva-derived biofilms. (**a**) Dynamic pH values of spent media for 6 days and (**b**) lactic acid production in the end of 6^th^ day. (**c**) Dynamic NH_3_ production of spent media for 6 days. All results are presented as mean ± standard deviation, each group was compared with the control group, saliva derived biofilm cultured with SHI medium, at the corresponding time point (n = 5; *p < 0.05; **p < 0.01; ***p < 0.001; ****p < 0.0001; ns, not significant).

The dysbiosis between acid-producing and alkali-producing bacteria raised the question of whether the acid and alkali metabolism of oral biofilms could be changed more significantly by sucrose than by other sugars. By quantifying the lactic acid and ammonia generation of biofilms receiving different sugar treatments, we found that the most significant disequilibrium between acid and alkali production was observed in sucrose-treated biofilms. The pH of the control group decreased to plateau at approximately 4.18; however, the pH values continuously decreased to 4.15 in the lactose group, 4.08 in the glucose group and 4.05 in the sucrose group (Fig. 6a). A similar trend was detected in lactic acid production on the 6^th^ day, as sucrose significantly enhanced the lactic acid production of the saliva-derived biofilm, while glucose and lactose did not (Fig. 6b). In addition, NH_3_ production was significantly suppressed by sucrose and to a lesser degree by glucose. However, no difference was observed in the lactose treatment group compared to the control (Fig. 6c).

## Discussion

Sucrose has long been considered the most cariogenic carbohydrate. In past decades, numerous studies have been carried out to understand the cariogenicity of sucrose, and these studies mainly focused on the effects of sucrose on the biochemical and physiological characteristics of dental biofilms favoring caries initiation and progression^12,28,31–33^. According to the ecological plaque hypothesis, caries results from an unfavorable shift in the resident oral microbial community (that is, a dysbiotic oral ecosystem) driven by environmental factors^2^. However, to the best of our knowledge, few studies have attempted to understand the microecological regulatory effects of sucrose on dental biofilms, and it is still unknown whether the prominent cariogenicity of sucrose is due to this macromolecule imposing higher ecological pressure on oral biofilms than other dietary sugars.

In the present study, we used the human salivary microbiome of healthy donors and SHI medium to prepare an *in vitro* multispecies biofilm model to test whether sucrose can introduce microecology imbalance favoring caries progression to a greater degree than the other sugars. Although this *in vitro* biofilm model system has been proven to maintain a highly reproducible species and metabolic diversity approaching that of the human oral microbiome^41,42^, we admitted that there is still some difference between our biofilm model and the *in vivo* dental biofilm because the diversity of the salivary microbiome may be reduced during centrifugation and some of the salivary bacteria are uncultivable even in SHI medium. The saliva-derived biofilm was cultured with SHI medium for 3 days, and then different sugars at the same concentration (2%) were added into the SHI medium to simulate the ecological challenges that the oral microbial community of healthy individuals faces. We noticed that, demineralization can be also detected in the control group (SHI medium alone), probably due to the fact that SHI medium contained 0.5% sucrose (which is an important ingredient to achieve a better recovery of the streptococci from the salivary samples)^41^, and that the biofilms were cultured in a closed system (i.e., a 24-well plate) which did not allow transfer of acid generated by bacteria through sugar metabolization out of the system. Nonetheless, we found that, all dietary sugars we supplemented into SHI medium enhanced the demineralization of the tooth enamel in comparison to SHI medium alone, among which sucrose caused the greatest demineralization lesion depth and most obvious mineral loss on enamel blocks, followed by glucose and then lactose. This result was consistent with the data of previous *in vivo* animal studies and epidemiologic studies^28,43,44^ showing that consumption of sucrose strongly influenced the incidence of dental caries and suggested that the microbiota changes we observed in the *in vitro* biofilm model here provided us with the chance to understand what happened *in vivo*. There seemed to be no linear dose–response relationship between sucrose supplementation and demineralization, as when 4 times more sucrose was added to the SHI medium than it was present in the control, only approximately 50% additional demineralization occurred. Potential reasons included, first, sugars were supplemented into SHI medium with high dosage. Since SHI medium contains 0.5% sucrose, the extra added sugars cannot be metabolized completely by bacteria. Therefore, we did not detect linear dose/response relationship between sucrose supplementation and demineralization. Second, previously published epidemiological data showed that neither a linear nor a log-linear relationship existed between sugar consumption and caries^45,46^. In that case, this finding from our *in vitro* experiment reflected what happened *in vivo*. Only a pooled salivary microbiome from healthy subjects was used to prepare biofilms in this study, and further studies using the salivary microflora from both healthy and caries-active subjects to establish an *in vitro* multispecies model and to compare whether cariogenic and noncariogenic dental biofilms have similar ecological responses to sugar challenge will be helpful for us to gain additional information about the etiology of caries.

To comprehensively understand the impact of different dietary sugars on the oral microecology, we profiled taxa within biofilms by using 16 S rRNA sequencing. Interestingly, no difference was detected in the microbial richness (represented by the number of OTUs) between sugar-treated groups and the control group, and we did not find sugar-specific OTUs, suggesting that if microbial structure shifts occurred after sugar exposure, they mainly resulted from the changed microbial relative abundances. Previously published *in vivo* data also showed that sugars mainly influence the microbial relative abundance but not the presence of the microflora, which is highly consistent with our findings and the ecological plaque hypothesis emphasizing the synergy of multiple bacterial species in the development of caries^47–50^. Therefore, we used weighted PCoA instead of unweighted PCoA analysis to compare the overall microbial structure between groups. As we expected, sucrose was the only tested sugar that significantly reshaped the overall microbial structure of oral biofilms. Ecological network analysis, a system-level method to identify species interactions within an ecosystem that cannot be directly observed^51^, further showed that sucrose-treated biofilms had no subnetworks, but there were more inter-modular connections in the sucrose network than in the glucose/lactose/control networks. Since the modules in the ecological networks could be regarded as putative microbial ecological niches or subcommunities^52^, the ecological network analysis suggested that sucrose treatment strengthened relationships between different oral bacterial community “niches”. In addition, the interspecies antagonism and synergy among microbiota within a microecological region is among the major mechanisms maintaining microbial homeostasis in dental biofilms^7^. Through ecological network analysis, we also found that the antagonistic relationships, which could be detected in the glucose/lactose/control groups, were absent from the microbial network of sucrose-treated biofilms, suggesting that sucrose influenced the interspecies interactions within the biofilms.

To look closely at how the microbial distribution was changed, we pinpointed the differently distributed microbiota between biofilms treated with sugars and the control. Again, we found that sucrose imposed the highest pressure on microbial taxa distribution. Some of the genera whose relative abundance was increased by sugar treatment in the present study, particularly *Gemella* and *Granulicatella*, were reported to be dominant in the saliva and dental biofilms of caries-active subjects^50,53,54^. Interestingly, *Veillonella*, one of the most prevalent genera in the human oral cavity^50,55^, showed lower relative abundance in only the sucrose-treated biofilms in comparison to the control, and the relative abundance of all species belonging to this genus detected in this study was significantly decreased by sucrose treatment. It was reported that the load of *Veillonella* in the caries-active adult population is lower than that of caries-free adults^29,53,54,56,57^ and that *Veillonella* reduces the risk of caries by converting lactate to less-acidic acids^34,35,58^. These data suggest that sucrose is more efficient at reducing the level of bacteria contributing to acid reduction within biofilms than glucose and lactose are. Considering that some species with very low abundance are hard to capture by 16 S rRNA sequencing, we further used qPCR to test the distribution of some well-known bacteria highly associated with caries (including the acid-producing species *S. mutans* and the alkali-producing species *S. sanguinis* and *S. gordonii*) within the biofilms to assess the homeostasis disruption between acid-producing and alkali-producing bacteria, as the enrichment of acidogenic pathogens and depletion of alkali-generating commensal residents within the biofilm is the core of dental caries pathogenesis^7–9^. We found that sucrose changed not only the bacterial load of all three species but also the ratio between acid-producing and alkali-producing bacteria. Sucrose enhanced both *S. mutans*/*S. sanguinis* and *S. mutans/S. gordonii* ratios, breaking the balance between acid-producing and alkali-producing bacteria within biofilms. Parallel to the dysbiosis between acid-producing and alkali-producing bacteria, the most significant disequilibrium between acid and alkali metabolism was detected in the sucrose group, as enhanced lactic acid production and suppressed ammonia production, which contributes to an environment with low pH that directly contributes to caries progression, were observed.

Taken together, our data demonstrated that the potential of sucrose in promoting the cariogenicity of oral biofilms was attributed to its ability to influence the microbial structure and assembly of the oral microecology and to disrupt the homeostasis between acid-producing and alkali-producing bacteria. These findings advance our knowledge of the mechanisms by which sucrose promotes dental caries formation from an ecological perspective.

## Materials and Methods

### Saliva collection

The present study was reviewed and approved by the Institutional Review Board of West China Hospital of Stomatology, Sichuan University (WCHSIRB-D-2017-095). Written informed consent was obtained according to the Ethical Guidelines of the Declaration of Helsinki (2014) before sampling^59^. Dental examination was carried out by one experienced dentist, and all enrolled volunteers (n = 10, aged 25~30) had no clinical symptoms of either oral or systematic diseases. Exclusion criteria also included body mass index ≥35 or ≤18, blood pressure >160/100 mmHg, oral temperature >37.8 °C, pulse >100 times/min, systemic medicine use in the last six months, local oral medicine use or treatment within 7 days, irregular eating within 3 months, and pregnant and lactating women. Subjects were asked to refrain from any food or drink 2 h before donating saliva. Approximately 5 ml of spontaneous, unstimulated whole saliva was expectorated into a sterile 15 ml cryogenic vial. Saliva samples were pooled together and then centrifuged at 2600 g for 10 min to spin down large debris and eukaryotic cells. The supernatant, referred to as pooled saliva, was diluted in sterile glycerol (final concentration of 25%), aliquoted and stored at −80 °C^41,42^. Some of the pooled saliva was further filtered using 0.22 μm filters. Filtered cell-free saliva was used to coat enamel blocks before growing *in vitro* multispecies biofilms^41,42^.

### Enamel block preparation

Molars were collected from patients who underwent third molar extraction surgery in the Department of Oral and Maxillofacial Surgery, West China Hospital of Stomatology, Sichuan University. Written informed consent was obtained before molar collection. Dental examination was carried out by one experienced dentist, and twenty-five molars without white spots, cracks or other defects were collected and stored with 0.05% thymol solution at 4 °C. Crowns were separated from roots and then cut into four sections (5 mm × 5 mm × 3 mm). The enamels were embedded in polymethylmethacrylate and then polished with water poor SiC abrasive papers (800~2400 grit; Struers, Struers Co., Ltd., Copenhagen, Denmark). The surface microhardness (SMH) of the sound enamel was determined with a microhardness tester (Duramin-1/−2; Struers), and five indentations spaced 100 μm apart were made on every exposed enamel surface at a 200 g load for 15 s. Enamel blocks with SMH between 320 and 400 Knoop hardness were selected for further study. After microhardness testing, the surfaces were slightly polished again. Each enamel surface was painted with two layers of acid-resistant varnish, leaving a 4 mm × 3 mm window. After ultrasonic cleaning in a deionized water bath for 10 min, the enamel blocks were sterilized in an ethylene oxide sterilizer.

### SHI medium preparation

To support the growth of different subpopulations within the pooled saliva, we prepared SHI medium and used it to culture biofilms. The composition of SHI medium was 10 g/l proteose peptone (Difco, Becton Dickinson, Sparks, MD, USA), 5.0 g/l trypticase peptone (Difco), 5.0 g/l yeast extract (Difco), 2.5 g/l mucin (type III, porcine, gastric; Sigma-Aldrich, St. Louis, MO, USA), 10 mg/l N-acetyl muramic acid (NAM, Sigma-Aldrich), 5% sheep blood, 2.5 g/l KCl, 5.0 g/L sucrose, 5.0 mg/l hemin, 1.0 mg/l vitamin K, 0.06 g/l urea and 0.174 g/l arginine^41^.

### *In vitro* saliva-derived multispecies biofilm culturing

Enamel blocks were put into 24-well plates, and 200 μl of filtered cell-free saliva was added to each well, allowing attached pellicle growth, and incubated at 37 °C with the lid open for 1 h to dry the saliva coating. The plates were then sterilized under UV light for 1 h before adding SHI medium (1.5 ml per well) and pooled saliva (30 μl per well). The plates were incubated at 37 °C without disturbance for the first 24 h under anaerobic conditions to allow initial biofilm formation. After 24 h, the culture medium was changed daily.

### Dietary sugar treatment

Starting at 72 h, the biofilms were transferred to SHI medium containing different dietary sugars (i.e., 2% sucrose, 2% glucose and 2% lactose) to simulate a cariogenic challenge until the endpoint (144 h). Figure 1 gives a brief introduction to the experimental design.

### Acid and alkali production measurement

The pH value of spent medium was measured with a pH meter (Thermo Fisher Scientific, Waltham, MA, USA), and the NH_3_ level in the medium was determined with an ammonia assay kit (Jiancheng, Jiancheng Bioengineering Institute, Nanjing, China). To measure the production of lactic acid in the 144 h biofilms, samples were rinsed in cysteine peptone water first and then transferred into new plates. Buffered peptone water (BPW) with 0.2% sucrose was added into each well and anaerobically incubated at 37 °C for 3 h. Lactic acid was determined by an enzymatic method as described before^60^. Briefly, the BPW solution absorbance was measured at 340 nm, and the lactic acid content was calculated based on a standard curve. The data are reported as the mean of 5 separate tests.

### Caries lesion depth and mineral loss assay

The caries lesion depth and mineral loss were measured by transversal microradiography (TMR). After removing the biofilms on the surface, enamel blocks were ultrasonically cleaned for 10 min. Then, blocks were cut into sections approximately 300 μm thick vertically to a window on the surface. Sections were then polished to a thickness of 100 μm. The polished slices were then fixed on a TMR sample holder with an aluminum calibration step wedge serving as a standard curve and microradiographed using a monochromatic CuK X-ray source (Philips, VitalAir/Comcare, Eindhoven, the Netherlands) at 20 kV and 20 mA for 30 s. The lesion depth and mineral loss at the selected area were analyzed by imaging software (Transversal Microradiography Software 2006, Inspektor Research Systems BV, Amsterdam, the Netherlands). Five TMR traces were measured on each slice. Mineral loss of the lesions was calculated by a computer program using a step wedge scale to compare the lesion to sound tissue. The lesion depth was defined as the length from the enamel surface to the point where the mineral content reached 95% of that of sound enamel. The data are reported as the mean of 5 separate tests, and representative pictures are shown.

### DNA extraction and 16 S rRNA sequencing

The biofilms were scraped with a sterile knife from enamel blocks into 1 ml of phosphate-buffered saline (PBS) and then sonicated. Genomic DNA was isolated using a QIAamp DNA micro kit (Qiagen, Valencia, CA, USA) according to the instructions but with additional lysozyme treatment (3 mg/ml, 1.5 h). The DNA quality was evaluated with a NanoDrop 2000 spectrophotometer (Thermo Fisher Scientific). Sequencing of the 16S rRNA amplicon (V1-V3 region) was performed using a MiSeq300PE (Illumina MiSeq System) at Majorbio Bio-pharm Technology Co.,Ltd (Shanghai, China). The primers used in the present study were 27 F (5′-AGAGTTTGATCCTGGCTCAG-3′) and 533 R (5′-TTACCGCGGCTGCTGGCAC-3′).

### Bioinformatic analysis of sequencing data

The sequences were clustered into 22 OTUs at the 97% similarity level. Bioinformatics was performed by mothur and QIIME 2.0 software, including quality control of raw data and taxonomic annotation according to the Silva database. The data were further analyzed as follows: (1) The microbial richness revealed by OTU number was analyzed with one-way ANOVA, followed by Dunnett’s t test to compare the means of all other groups with that of the control group. (2) A Venn diagram was constructed to compare the shared and different OTUs among groups. (3) Weighted PCoA and dissimilarity tests, including Adonis and ANOSIM, were used to examine the community differences between groups. (4) Microbe-microbe networks were constructed by the MENA pipeline. A Pearson correlation cutoff of 0.7 was determined using the random matrix theory approach by observing the transition point of the nearest-neighbor spacing distribution of eigenvalues from a Gaussian to a Poisson distribution, two universally extreme distributions. Then, Cytoscape 3.2.0 was performed with a force-directed algorithm to visualize networks and compute network topological parameters with NetworkAnalyzer^47^. (5) The relative abundances of bacterial taxa at the genus and species levels were calculated. The relative abundance difference between groups was compared by the Kruskal-Wallis test at both the genus and species levels.

### Microbial quantification by qPCR

*S. mutans*, *S. sanguinis*, *S. gordonii* and *V. parvula* were quantified using qPCR with standard curves as previously described^10,61^. Primers were chosen based on the bacterial 16 S rRNA sequences (Table 1). qPCR amplification was performed on the Applied Biosystems 7500 Real-Time PCR System (Thermo Fisher Scientific). The reaction mixture (20 μl) contained 10 μl of 2 × SYBR *Premix Ex Taq II* (TaKaRa, Takara Bio Inc., Shiga, Japan), 2 μl of template DNA, 0.8 μl of forward and reverse primers (10 mM each), 0.4 μl of ROX Reference Dye II and 6 μl of PCR-grade water. Thermal cycling conditions were as follows: initial denaturation at 95 °C for 30 s, followed by 40 cycles of 95 °C for 5 s and 60 °C for 34 s. An additional step, 95 °C for 15 s, 60 °C for 1 min and 95 °C for 15 s, was performed to establish a melting curve. Threshold cycle values (CT) were determined, and the copy number was calculated based on the standard curve (log copies/μl versus CT values). Each sample was examined in triplicate. The data are reported as the mean of 5 separate tests.

**Table 1:** Oligonucleotide primers used in qPCR for microbial quantification.

Species	Primer name	Sequence(5′—3′)
*S. mutans*^62^	*S. mutans*-F	GATAATTGATTGAAAGATGCAAGC
	*S. mutans*-R	ATTCCCTACTGCTGCCTCCC
*S. sanguinis*^63^	*S. sanguinis*-F	AGTTGCCATCATTGAGTTG
	*S. sanguinis*-R	GTACCAGCCATTGTAACAC
*S. gordonii*^63^	*S. gordonii*-F	GCTTGCTACACCATAGACT
	*S. gordonii*-R	CCGTTACCTCACCTACTAG
*V. parvula*^64^	*V. parvula*-F	TGCTAATACCGCATACGATCTAACC
	*V. parvula*-R	GCTTATAAATAGAGGCCACCTTTCA

### Statistical analysis

Data other than those obtained from microbiome sequencing were compared with SPSS (version 16.0 for Windows). Data were analyzed with one-way ANOVA, followed by Student-Newman-Keuls test to compare means of each group, and Dunnett’s t test to compare the mean of all other groups with the control group. The difference was statistically significant if the 2-tailed p value < 0.05.

## Data Availability

All data generated or analyzed during this study are included in this published article. The 16 S rRNA sequencing raw data have been deposited in the public database Sequence Read Archive (http://www.ncbi.nlm.nih.gov/Traces/sra) under accession number PRJNA550154.
